# Can we decrease the duration of basal thumb joint distraction for early osteoarthritis from 8 to 6 weeks? Study protocol for a non-inferiority randomized controlled trial

**DOI:** 10.1186/s13063-021-05283-9

**Published:** 2021-05-01

**Authors:** Janna S. E. Ottenhoff, Teun Teunis, Assa Braakenburg, Aebele B. Mink van der Molen

**Affiliations:** 1 Department of Plastic, Reconstructive and Hand Surgery, University Medical Center Utrecht, Heidelberglaan 100, 3508 GA Utrecht, The Netherlands; 2 Department of Plastic, Reconstructive and Hand Surgery, St. Antonius Hospital, Koekoekslaan 1, 3435 CM Nieuwegein, The Netherlands

**Keywords:** Osteoarthritis, Thumb, Carpometacarpal Joint, External fixator, Complications, Treatment

## Abstract

**Background:**

To our knowledge, to date, 52 patients with thumb carpometacarpal osteoarthritis (CMC1 OA) were treated with joint distraction. So far, most patients experienced improved physical function and less pain. After 2 years, only 1 patient proceeded to trapeziectomy. This study assesses if we can safely lower the distraction duration from 8 to 6 weeks for CMC1 joint distraction, maintaining the improvement in physical function and pain.

**Methods:**

This is a monocenter randomized controlled non-inferiority trial that includes patients younger than 65 years of age with ongoing symptoms of CMC1 OA and an established indication for surgery. All patients will be treated with CMC1 joint distraction. The primary outcome is to assess whether 6 weeks of joint distraction is not inferior to 8 weeks in terms of physical function at 1 year after surgery. Secondary outcomes will identify differences between groups at 1 year in pain intensity, patient satisfaction, hand health status, adverse event rates, treatment failure, differences in thumb strength and range of motion, and radiographic changes.

**Discussion:**

If safe, the duration of basal thumb joint distraction can be reduced to 6 weeks, reducing patient burden. Because this is a relatively new treatment, this trial will provide greater knowledge of potential adverse events. This knowledge allows for more informed decision making for patients considering CMC1 distraction treatment. Future studies can directly compare joint distraction to other treatments of CMC1 joint arthritis like splinting and trapeziectomy.

**Trial registration:**

Central Committee on Research Involving Human Subjects (CCMO), NL68225.100.18; registered on 9 August 2019. Medical Research Ethics Committees United (MEC-U), R19.003; registered on 9 August 2019. Netherlands Trial Register, NL8016; registered on 15 September 2019.

**Supplementary Information:**

The online version contains supplementary material available at 10.1186/s13063-021-05283-9.

## Background

One out of three people aged 55 years and older has radiographic signs of carpometacarpal osteoarthritis of the thumb joint (CMC1 OA) [1–3]. The prevalence increases with ageing to 90% for adults aged 80 years and older [1–3]. Patients with symptoms of CMC1 OA are initially offered non-operative treatment including splints, analgesics, and hand therapy to reduce pain [4]. If these interventions do not offer sufficient relief, surgical treatment can be considered [4–6]. There are numerous variations in surgical treatment for CMC1 OA. There is, however, no evidence for the superiority of individual techniques regarding pain and functional outcome [5–7]. Trapeziectomy alone, or combined with ligament reconstruction and tendon interposition or suspension arthroplasty, carries the long-term risk of metacarpal subsidence with or without persisting symptoms [5–9]. Prostheses are associated with loosening, subluxation, fracture, and synovitis, potentially requiring revision surgery [5–10]. Arthrodesis of the CMC1 joint reduces range of motion and has the associated risk of non-union resulting in revision surgery [11]. For patients with persisting symptoms of CMC1 OA requiring surgical intervention at a relatively young age, other techniques that preserve the joint and are less invasive may be more desirable.

Joint distraction is a joint sparing treatment for relatively young patients (< 65 years of age) with symptoms of OA and aims to postpone or prevent an invasive surgical intervention [12–15]. Previous evidence on ankle and knee OA shows that joint distraction can result in sustained clinical improvement with actual repair of joint cartilage after treatment [12, 14–18]. Van der Woude et al. [15] showed persisting pain reduction and greater physical function compared to baseline at 5 year follow-up among patients who underwent knee distraction. Another study demonstrated an almost 50% (8/17) joint survival rate after 9 years [14].

Joint distraction is also feasible for the osteoarthritic CMC1 joint [19]. Nowadays, to our knowledge, more than 50 patients with persisting symptoms of CMC1 OA despite non-operative therapy were treated with CMC1 joint distraction. Recent follow-up results of 20 patients who underwent CMC1 joint distraction shows that in 19 of 20 patients an invasive surgical intervention was postponed for at least 2 years (unpublished data). On average, all patients experienced less pain and better physical function after 2 years. Unpublished data of the first 5 patients shows that all patients were still satisfied 5 years post-distraction without further surgical interventions.

Distraction of the CMC1 joint is currently applied for 8 weeks. However, the duration of knee joint distraction has been decreased from 8 to 6 weeks [17]. This is based on results of 2 previous studies that report similar clinical results at 1 year and less pin tract infections among patients in the 6-week group [17, 18]. Pin tract infections occurred in 85% of patients in the 8-week group versus 55% in the 6-week group [17]. During CMC1 joint distraction, pin-tract infections occur in approximately 1 of 3 patients and are usually adequately treated with oral antibiotics (unpublished data of 40 patients). It is unknown if shortening of the CMC1 joint distraction duration from 8 to 6 weeks will also result in less adverse events and still achieve sufficient clinical benefits for patients. Therefore, we designed this study protocol for a randomized controlled non-inferiority trial to compare 6 weeks with 8 weeks of CMC1 joint distraction.

### Study objectives

The primary objective is to assess if 6 weeks of CMC1 joint distraction is not inferior to a duration of 8 weeks. Our primary outcome is physical function measured with the Patient-Reported Outcomes Measurement Information System Physical Function for the Upper Extremity (PROMIS UE) at 1 year after distraction.

Our secondary objectives are:

- We hypothesize that there is no difference between groups in terms of pain intensity, patient satisfaction, joint stiffness, thumb function, range of motion, and strength at 1 year.
- We hypothesize that there is no difference in hand health status measured with the Michigan Hand Outcome Questionnaire (MHQ) between the 6-week and 8-week group at 1 year.
- We will investigate if there is a difference in adverse event rate and treatment failure at 1 year.
- We will measure minimal joint space width on radiographs at 1 year and test if there is a difference between the two groups.
- We will assess symptoms of depression and catastrophic thinking (captured by 2 short questionnaires) since there is mounting evidence that psychosocial factors influence symptom intensity and magnitude of physical limitations among patients with CMC1 OA [20–22]. We will test if these, and other factors, are independently associated with physical function and hand health status.
- To study the long-term effects of joint distraction in the treatment of CMC1 OA, we will test all hypotheses mentioned above at 2 years and 5 years post-distraction.

## Methods/design

### Study design

This is a monocenter randomized controlled non-inferiority trial conducted at the St. Antonius Hospital in the Netherlands: a peripheral teaching hospital in a large urban area. Patients will be randomly assigned to group A or B using a secured computer programme (Research Electronic Data Capture (REDCap)). All patients are treated with continuous CMC1 joint distraction by placing an external distractor device over the affected joint. The distractor device will be removed after 6 weeks among patients in group A; for patients randomized to group B, the device is removed after 8 weeks.

#### Participants

The study population consists of 68 patients with ongoing symptoms of CMC1 OA despite non-operative treatment. All patients have an established indication for an invasive surgical intervention (such as a trapeziectomy) at a relatively young age (< 65 years).

#### Inclusion criteria

In order to be eligible to participate in this study, patients must meet all of the following criteria:

- Age < 65 years
- Eaton-Glickel classification II or III on radiographs [23]
- Failed non-operative treatment (e.g. splint for at least 3 months)
- Established indication for invasive surgical treatment for CMC1 OA according to standard clinical practice
- Willingness to participate in the study and able to understand distractor maintenance and hygiene instructions

#### Exclusion criteria

Patients who meet any of the following criteria will be excluded from participation in this study:

- Severe CMC1 OA with Eaton-Glickel grade IV on radiographs [23]
- Joint subluxation of > 30%
- Surgical treatment of the CMC1 joint in the past
- Inflammatory or rheumatoid arthritis present or in past medical history
- Use of immunosuppressive or chemotherapeutic drugs
- Previous corticosteroid injection in the CMC1 joint
- Hypermobility syndrome or syndromic diseases
- Unable or unwilling to attend follow-up appointments

### Surgical procedure

**Fig. 1:**
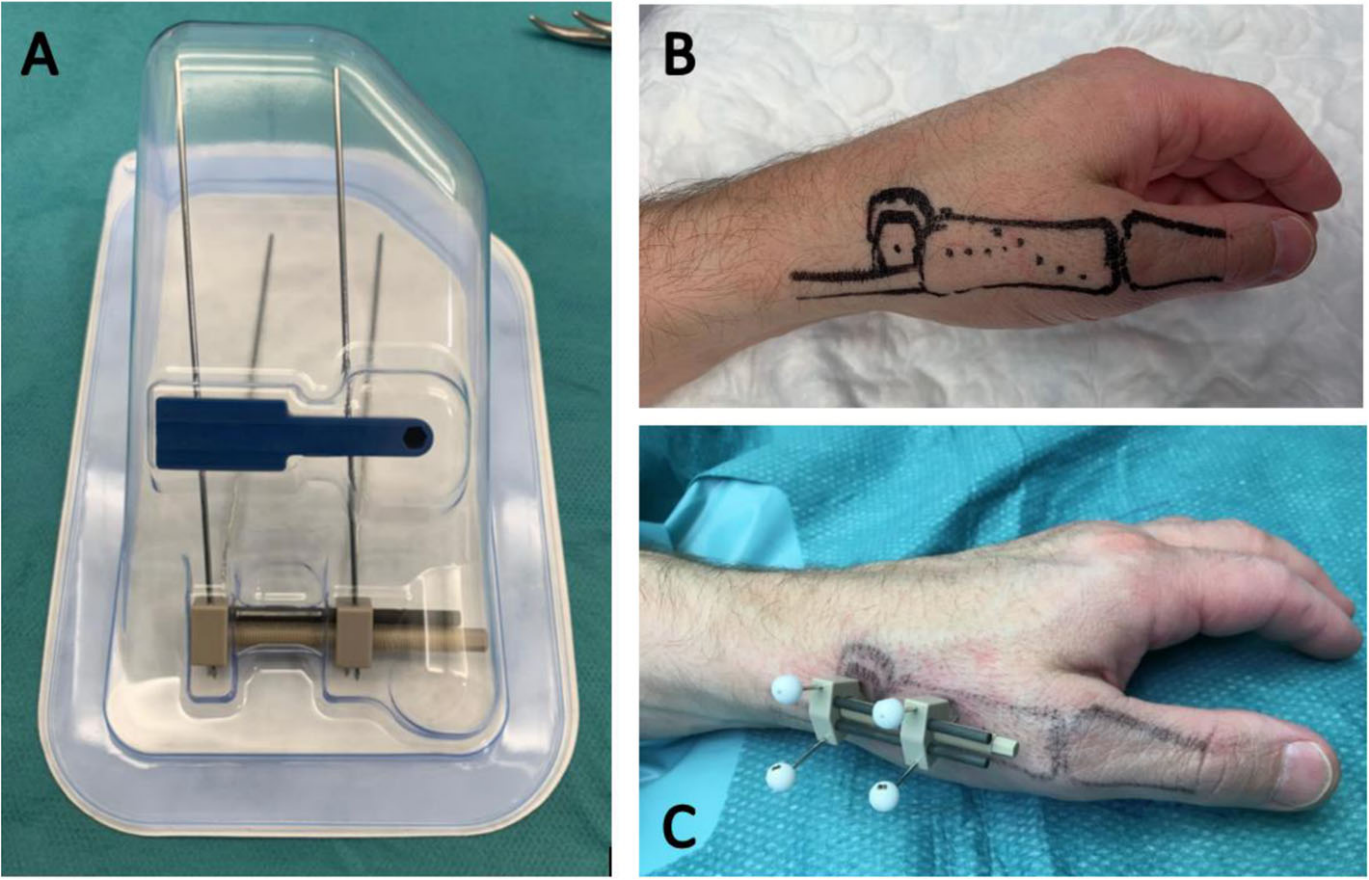
**a** The distractor device. **b** Drawing of the trapezium bone and metacarpal bone. **c** Distractor device in situ with 2 k-wires in the trapezium and 2 in the metacarpal bone

**Fig. 2:**
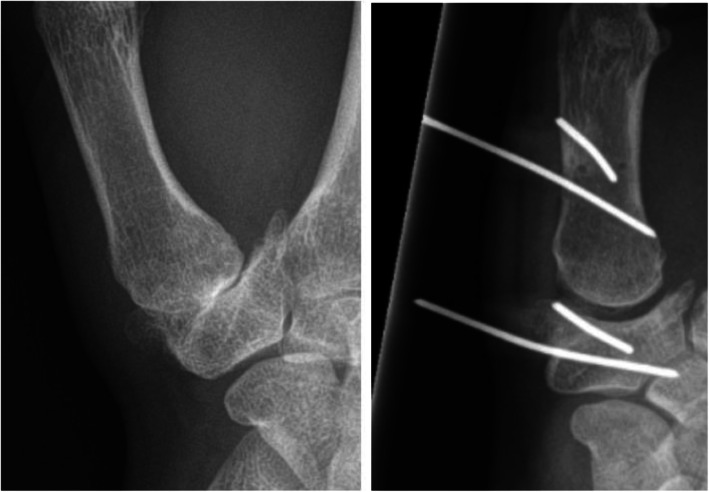
Radiograph of the CMC1 joint before (left) and during distraction (right). Published with permission of the original authors and the Journal of Plastic Surgery and Hand Surgery www.tandfonline.com (reference 19)

**Fig. 3:**
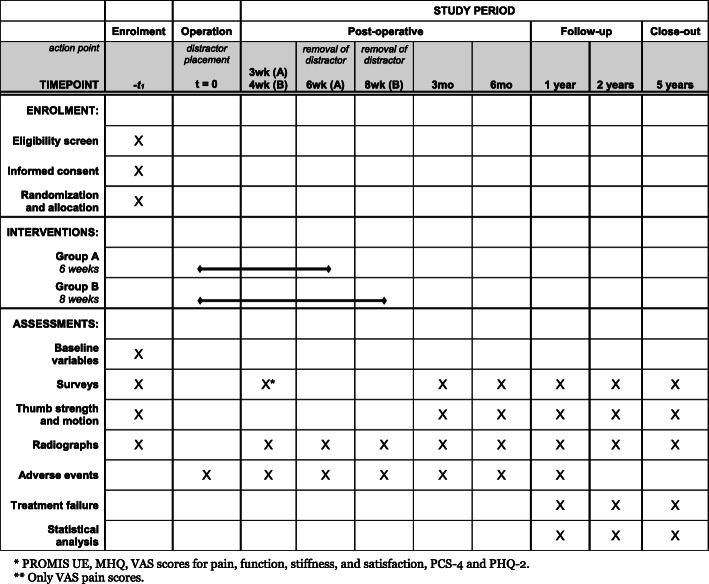
Standard Protocol Items: Recommendations for Interventional Trials (SPIRIT) figure showing the phases of the trial and data collection time points

CMC1 joint distraction will be performed by one of two hand surgeons with experience in this procedure. Both surgeons are hand fellowship trained and certified by the Federation of the European Societies for Surgery of the Hand. They have developed and performed all CMC1 joint distractions (*n* = > 60) since the start of this treatment in 2014. Their level of experience is a category 5— according to the classification by Jin Bo Tang—based on the pioneering contribution both surgeons made in developing this technique [24]. Patients will be operated under regional anaesthesia, unless patients prefer general anaesthesia. All patients receive systematic antibiotics perioperative (2 g cefazoline intravenous). An external distractor device (Osteo-x, Osteotec, Dorset, UK) is placed over the affected CMC1 joint (Fig. 1a). The device is anchored transcutaneous with 2 proximal k-wires in the trapezium bone and 2 distal k-wires in the first metacarpal bone (Fig. 1b). Subsequently, the distractor device is distracted 3 mm intraoperative. The k-wires are cut 1.0 to 1.5 cm above the device (Fig. 1c). Adequate positioning of the distractor and proper placement of the k-wires in the trapezium and metacarpal bone is checked with video-fluoroscopy during the procedure. The position of the device is checked with standard radiographs at set postoperative intervals (Figs. 2 and 3). Patients are given a custom-made thermoplastic splint to cover and protect the distractor device. Hygiene instructions regarding pin entry point maintenance will be given. Patients are discharged after surgery (daycare), unless the unlikely event occurs that a hospital admission is needed. During the period of distraction, patients will be seen at the outpatient clinic at set post-operative intervals.

### Study parameters

#### Primary parameter

The primary parameter is physical function at 1 year measured with the Patient-Reported Outcomes Measurement Information System Physical Function for the Upper Extremity (PROMIS UE) [25]. This is a validated 16-item questionnaire answered on a 5-point Likert scale. The PROMIS UE has *T*-scores with a mean of 50, and higher scores indicate better physical function.

#### Secondary study parameters

- Patient characteristics: gender, age at operation, work status, marital status, and level of highest education.
- Michigan Hand Outcome Questionnaire (MHQ - Dutch Language Version): a validated 57-item questionnaire that gauges hand health status on a scale from 0 to 100 with higher scores indicating better hand health status [26].
- Patient Health Questionnaire (PHQ-2): 2-item questionnaire with scores from 0 representing ‘lowest level of depression’ to 6 ‘highest level of depression’ [27].
- Pain Catastrophizing Scale (PCS-4): 4-item questionnaire rated on a 5-point Likert scale with scores ranging from 0 indicating ‘no catastrophic thinking’ to 16 ‘worst possible catastrophic thinking’ [28].
- Visual analogue scale (VAS) scores for pain, patient satisfaction, thumb stiffness and function: with scores from 0 representing ‘no pain at all’ or ‘fully satisfied’ or ‘no stiffness at all’ or ‘optimal function’ to 100 ‘worst pain possible’ or ‘not at all satisfied’ or ‘worst stiffness possible’ or ‘worst possible function’.
- Range of motion obtained by an independent certified hand therapist:
- Thumb opposition by Kapandji scores (range 0–10)
- Palmar thumb abduction (degrees) by Pollexograph [29]
- Strength measures* (in kg) obtained by an independent certified hand therapist:
- Grip strength measured with a Jamar hand dynamometer
- Key and tip pinch strength measured with baseline pinch gauge
*Strength measures are recorded as the average of three attempts
- Radiographs will be obtained in three different views (posteroanterior, lateral and Bett’s view). Joint space (in mm) will be scored by an independent blinded radiologist.
- Adverse events: classified according to the Clavien-Dindo classification of surgical complications ranging from grade I (minor complication) to grade V (death) [30]. Any adverse event that occurs during the distraction period or at follow-up will be administered and classified. For example, pin tract infections that are adequately treated with oral antibiotics will be classified as grade I (minor complication). In the unlikely event that intravenous antibiotics and a hospital admission is needed, this will be rated as a grade II complication.
- Treatment failure: defined as conversion to an invasive surgical procedure (f.e. trapeziectomy) after distraction therapy due to ongoing symptoms of CMC1 OA.

### Study phases

#### Recruitment and consent

Patients visiting the plastic surgery outpatient clinic of the St. Antonius Hospital in the Netherlands due to symptoms of CMC1 OA will be screened for eligibility. A radiograph is taken at initial visit as standard of care. The hand surgeon will assess eligible patients for enrolment based on the set inclusion and exclusion criteria. Patients who fulfil the inclusion criteria will be invited to participate in the study. Written informed consent will be obtained by the coordinating investigator from all participants.

#### Preoperative measurements

Patients will be asked to complete a series of questionnaires on REDCap, a secured web-application for clinical research, including patient demographics (gender, age, marital status, etc.), physical function, pain scores, and symptoms of depression. All questionnaires are described in detail under ‘study parameters’. Thereafter, patients will visit the hand therapist for strength and range of motion measures of both hands. Next, placement of the distraction device is scheduled and patients will be seen by the anaesthesiologist for screening and approval of the surgical procedure.

#### Postoperative appointments and measurements

The study phases and data collection time points are shown in Fig. 3. The Standard Protocol Items: Recommendations for Interventional Trials (SPIRIT) checklist is provided as Additional file 1. In case of any problems or concerns regarding the distraction device, extra visits will be scheduled if needed.

After placement of the distractor device, a thermoplastic splint is applied and hygiene instructions are given. Patients can go home on the same day of surgery. It is not allowed to drive a car, lift objects or bear weight with the operated hand during the duration of distraction therapy. Three weeks (for group A) or 4 weeks (for group B) after placement of the distractor device, patients are seen at the outpatient clinic. Only VAS pain scores will be collected at this time point and no other questionnaires or measurements are assessed. Any adverse events will be registered if needed.

The distractor device will be removed at the outpatient clinic after 6 weeks for patients in group A and after 8 weeks for patients in group B. After removal, a radiograph is obtained and hand therapy commences according to a standard protocol for rehabilitation after surgical intervention. Patients are informed not to perform heavy weight bearing exercises of the thumb and index finger up to 12 weeks after removal of the distractor device. There is no other relevant concomitant care permitted or prohibited during the trial. A short overview of the exercises and timeline is provided in Additional file 2.

At 3 months, 6 months, and 1 year after placement of the distractor device (regardless of group), patients will be seen for follow-up measures at the outpatient clinic. At these visits, several questionnaires will be completed, radiographs of the thumb will be obtained, and any adverse events that may have occurred will be evaluated and registered. The same measurements are collected at 2 years and 5 years after initial surgery to evaluate the long-term effects of joint distraction. If patients underwent other surgical interventions for ongoing symptoms of CMC1 OA, despite joint distraction of the affected hand, this will be registered as treatment failure.

Patients will receive an invitation letter for their next follow-up appointment. We will call or mail those who do not follow-up and cannot be reached. Patients are always able to contact the investigator or the St. Antonius Hospital for any reason, question, or problem.

### Potential benefits and risks assessment

Joint distraction is a fairly new treatment for CMC1 OA. Patients will be informed that this treatment is not offered in regular clinical practice yet, only in the context of a formal clinical study. By participating in the study, patients contribute to better understanding in the place of CMC1 joint distraction therapy compared to the presently available surgical alternatives, which may be beneficial to patients in the future. Rehabilitation is not expected to be longer than that of the currently available invasive operative interventions.

#### Potential risks or complications

- Radiation burden. A total of eight radiographs will be obtained during the study period. The radiation burden will be 0.02 mSv per radiograph, resulting in a total amount 0.16 mSv. In our opinion, this is an acceptable small amount.
- Pin tract infections. Based on data from the previous cohort, pin tract infections occur in approximately 1 out of 3 patients. It can usually be adequately treated with oral antibiotics. In case of persisting infection, the distractor device may need to be removed at an earlier time point. In case of severe infection, hospital admission and intravenous antibiotics may be needed, but the estimated risk is minimal.
- Loosening of the device due to direct external forces. A customized thermoplastic splint is fashioned directly after placement of the device to provide cover and protection. If, for any reason, the device is loosened or dislodged, re-fixation or removal will follow varying per case.
- Disappointing outcome. In case distraction therapy yields insufficient improvement, the established options of invasive surgical treatment will still be possible, albeit with delay caused by study participation*.*

#### Potential benefits

- Patients may experience significant clinical benefits (less pain, better physical function) after this minimal invasive procedure.
- Patients in the 6-week group may experience less pin tract infections. A distraction period of 6 weeks has not been studied for CMC1 OA specifically, but based on knee distraction studies, 6 weeks of continuous joint distraction seems to result in less pin-tract infections compared to an 8-week distraction duration (85% versus 55% respectively) [17, 18]. However, it must be mentioned that pin tract infections occur more often during knee distraction than CMC1 joint distraction treatment [19].

### Randomization and blinding

Patients will be randomized to one of two groups at a 1:1 allocation ratio. We will use Research Electronic Data Capture (REDCap), a secure web-application and electronic platform for managing clinical research data [31], for randomization and allocation concealment. A fixed block size design will be generated in this secure web-application by the coordinating investigator who is not involved in patient care or in the assessment of the postoperative outcomes. Details about the randomization method or block sizes will not be available to or shared with those who enroll participants, assign interventions, or assess outcomes. Once a patient has been enrolled, the research assistant will log into the secured computer system (REDCap) and assign patients to group A or B. During placement of the distractor, the hand surgeon, operating room-assistant, and nurses will not know to which group the patient is randomized. Patients cannot be blinded. Radiographs will be scored by a blinded radiologist.

### Sample size calculation

We aim to assess non-inferiority of 6 weeks distraction compared to 8 weeks measured by PROMIS UE scores. We performed a sample size calculation based on the minimal clinically important difference (MCID) of the PROMIS UE questionnaire. The MCID is the smallest difference that patients perceive as beneficial. Previous studies report a MCID of 9.0 points for PROMIS UE with a standard deviation of 11 [32]. Non-inferiority will be considered if the mean difference with 95% confidence interval (95% CI) is no more than 9.0 points lower in the 6-week group compared to the 8-week group. To detect non-inferiority with 90% power, and a one-sided confidence level set at 97.5%, and 5% estimated loss to follow-up, we aim to include 68 patients. Each month, it is estimated that 4 patients will be eligible and willing to participate, resulting in an inclusion time of 2 years.

### Statistical analysis

The following characteristics will be reported in the baseline characteristics table: age, gender, work status, marital status, level of education, PROMIS UE scores, MHQ, PHQ-2, PCS-4, VAS for pain, satisfaction, thumb stiffness, thumb function, range of motion, strength, and joint space width on radiographs. Testing for differences in baseline characteristics among groups will only be done if visual inspection of the results indicates possible significant differences.

The primary outcome will be the difference in PROMIS UE score between the 6-week group (A) and 8-week group (B) at 1 year follow-up. We will report the mean with SD and 95% CI and the mean difference with 95% confidence interval. If the mean difference with 95% CI falls within the predefined non-inferiority margin of 9.0 points of PROMIS UE scores, we will conclude non-inferiority.

Regarding the secondary hypotheses, we will test for superiority between the 6-week and 8-week group.

- We will assess any differences in MHQ scores, VAS pain, VAS satisfaction, VAS function, VAS stiffness, range of motion, and strength at 1 year follow-up compared to baseline. We will test for superiority between groups by comparing the mean differences at 1 year.
- Treatment failure is scored when a patient proceeds with an invasive surgical procedure (f.e. trapeziectomy) after distraction therapy due to ongoing symptoms of CMC1 OA. We will analyse the difference between groups in the proportion of participants who are classed as treatment failure at 1 year.
- Adverse event rates will be reported and classified according to the Clavien-Dindo classification of surgical complications. We will analyse the difference between groups in the proportion of participants who are classed as adverse event at 1 year.
- Joint space width at 1 year follow-up is compared to baseline measures in both groups. Mean difference in mm between groups is assessed at 1 year follow-up.

This will be intention-to-treat analyses. In case of non-adherence, to test the robustness of our results, we will also report the results of per-protocol analyses [33].

We will create 2 multivariable linear regression models to assess factors independently associated with PROMIS UE and MHQ scores at 1 year. In these models, we will include all mentioned study parameters (except PROMIS UE and MHQ scores) with *P* < 0.10 in bivariate analysis. *P* values < 0.05 are considered statistically significant.

We will perform the same statistical tests and analysis as mentioned above at 2 years and 5 years post-distraction to study the long-term results. Incomplete data will be adequately described. We will use multiple imputation for any missing data or means for missing values.

### Withdrawal

Participants can leave the study at any time for any reason if they wish to do so, without any consequences. If a patient wishes to withdraw during the distractor period, the distractor can be removed in the outpatient clinic and standard treatment will continue. After removal of the distractor, a patient is also free to discontinue participation by refusing to complete questionnaires or follow-up imaging. We do not anticipate any circumstance in which the investigator would recommend the patient withdraws from the study since the potential risks and complications expected are limited and not life-threatening or harmful (see the section “Potential benefits and risks assessment”).

### Data management, monitoring, and publication

Data will be handled confidentially. Data will be administered on an encrypted computer in REDCap: a secured electronic data capture tool for clinical research [31]. Patient data will be anonymized. All included patients are identified by a patient identification number. A list of these numbers with name combinations will be securely stored by the coordinating investigator. The handling of personal data will be performed in compliance with the Dutch Personal Data Protection Act and in compliance with Good Clinical Practice guidelines. Data will be kept for 15 years after the end of the study. Written informed consent from study participants will securely locked away within the hospital.

This study will be monitored by a certified monitor according to the St. Antonius Hospital and Medical Research Ethics Committees United (MEC-U) monitoring policy. Insurance is provided for all participants in accordance with Dutch legislation. The results of this study will be published in peer-reviewed journals and presented at (inter)national conferences. Any protocol amendments will be submitted at the MEC-U for approval and relevant parties, including participants, will be informed if needed.

## Discussion

Joint distraction is a fairly new treatment for patients with CMC1 OA [19]. To our knowledge, to date, 52 patients have been treated with joint distraction. In contrast to other joint distraction treatments, CMC1 distraction is not offered in regular clinical practice yet, only in study context. Distraction therapy can result in less pain and better physical function and can therefore postpone an invasive surgical intervention [12–16, 18]. In a previous study about CMC1 joint distraction, a surgical intervention was postponed for at least 2 years in most (19 of 20) patients (unpublished data).

This trial will test if we can decrease the current distraction duration from 8 to 6 weeks and still achieve sufficient clinical benefits for patients. For knee distraction, a decreased treatment duration of 6 weeks (instead of 8 weeks) resulted in less pin tract infections while good clinical results were still achieved [17, 18]. Therefore, knee distraction is nowadays applied for 6 weeks. It is unknown if 6 weeks of continuous CMC1 joint distraction, compared to 8 weeks, leads to similar results: less adverse events and sufficient clinical benefits (e.g. less pain and better physical function). The outcomes of this study will give a more decisive answer to this question. If safe, the duration of basal thumb joint distraction can be reduced to 6 weeks, reducing patient burden.

This study will also enable to assess the short- and long-term effects of joint distraction in 68 patients. We expect that joint distraction will lead to less pain and better physical function in patients on average, regardless of group. Because this is a relatively new treatment, this trial will provide greater knowledge of potential adverse events. This knowledge allows for more informed decision making for patients considering CMC1 distraction treatment and will help to better define the place of joint distraction in treatment of CMC1 OA.

It is not feasible to blind participants to wearing a distractor for 6 or 8 weeks. Due to logistical constraints, we are also unable to blind surgeons and hand therapists. The lack of blinding might influence our results, but is common in trials assessing a surgical intervention. Besides, we realise the need for additional studies to compare joint distraction with other operative and non-operative techniques. However, we first designed this study to explore the possibilities to decrease distraction duration and bring CMC1 joint distraction, in this regard, in line with other joint distraction techniques. Based on the results of this current study, we will conduct a next comparative study to achieve a better understanding of the effects and benefits of joint distraction directly compared to other techniques (like splinting or trapeziectomy) in the treatment of CMC1 OA.

This study will mainly focus on clinical and patient-reported outcomes. We realise that there is also a need to gain more knowledge about the working mechanism of joint distraction. Future studies can contribute to a better understanding of this mechanism by—for example—focusing on arthroscopic sampling of articular cartilage, detailed imaging techniques, or biochemical analysis of synovial fluids.

There is major evidence that psychosocial factors—such as catastrophic thinking and symptoms of depression and anxiety—account for more of the variation in CMC1 OA symptom intensity than measure of pathophysiology [20, 21, 34]. To study differences in the magnitude of psychosocial factors before and after CMC1 joint distraction, in this study, we will measure symptoms of pain catastrophizing and of depression with two short questionnaires (PHQ-2 and PCS-4) [27, 28]. This could lead to a better understanding of the impact of psychosocial factors on physical function and other outcomes after CMC1 joint distraction therapy. Future studies can focus on exploring other/additional treatment opportunities for patients with CMC1 OA to optimize care (f.e. more effective coping strategies). This could result in a more multidisciplinary approach in treatment of CMC1 OA.

## Trial status

This study was registered at the CCMO (Central Committee on Research Involving Human Subjects) in the Netherlands on 9 August 2019 (NL68225.100.18), at the Medical Research Ethics Committees United (MEC-U) on 9 August 2019 (R19.003), and registered with the Netherlands Trial Register (registration number NL8016) on 15 September 2019. This manuscript is based on research protocol version number 3.0, dated 24 July 2019. Recruitment started at the St. Antonius Hospital, the Netherlands on 5 December 2019. The approximate date on which recruitment will be completed, is 31 December^,^ 2021.

## Supplementary Information

**Additional file 1.** SPIRIT Checklist.

**Additional file 2.** Hand therapy protocol.

**Additional file 3.** Ethical approval document (in Dutch).

**Additional file 4.** English translation of Add 2.

**Additional file 5.** Copy of original funding document.

**Additional file 6.** English translation of Add 4.

**Additional file 7.** Provisionally permission for re-use of figures.

## Data Availability

Data sharing is not applicable to this article; no datasets will be available during the current study.

## Abbreviations

CCMO: Centrale Commissie Mensgebonden Onderzoek
95% CI: 95% confidence interval
CMC OA: Carpometacarpal osteoarthritis
MCID: Minimal clinically important difference
MEC-U: Medical Research Ethics Committees United
MHQ: Michigan Hand Outcome Questionnaire
PCS-4: Pain Catastrophizing Scale-4
PHQ-2: Patient Health Questionnaire-2
PROMIS UE: Patient-Reported Outcomes Measurement Information System for Physical Function of the Upper Extremity
REDCap: Research Electronic Data Capture
VAS: Visual analogue scale
