# Supplementary material for: Can we decrease the duration of basal thumb joint distraction for early osteoarthritis from 8 to 6 weeks? Study protocol for a non-inferiority randomized controlled trial
Source: Trials. 2021 May 1;22:316. doi: 10.1186/s13063-021-05283-9 (PMC8088687; doi:10.1186/s13063-021-05283-9)
Supplement: Supplementary file 2 — Additional file 2. Hand therapy protocol. [file 13063_2021_5283_MOESM2_ESM.docx]

**HAND THERAPY PROTOCOL AFTER CMC1 JOINT DISTRACTION**

**After distractor removal (6 or 8 wks after surgery)**

- Start with motion exercises:
  - Active thumb opposition
  - Active thumb palmar abduction
  - Active thumb extension
  - No weight bearing exercises with thumb and index finger yet.
- Advice for daily activities:
  - Only use thumb for light activities, no heavy lifting yet.
  - Driving a car is allowed if a patient feels safe to do so.
- Follow-up appointments are scheduled depending on thumb stiffness, pain and patient preference.
  - If the thumb is very stiff and painful: weekly visits are scheduled.
  - Otherwise the next visit is scheduled after two weeks and every other week thereafter.

**Two weeks after removal of distractor (8 or 10wks after surgery)**

- Continue motion exercises, expand if possible.
- Advice for daily activities:
  - Use thumb for moderate activities, no heavy lifting yet.
  - Riding a bike is allowed if a patient feels safe to do so.

**Three months after placement of distractor**

- Advice for daily activities:
  - All activities are allowed, including lifting.
