# Supplementary material for: Can we decrease the duration of basal thumb joint distraction for early osteoarthritis from 8 to 6 weeks? Study protocol for a non-inferiority randomized controlled trial
Source: Trials. 2021 May 1;22:316. doi: 10.1186/s13063-021-05283-9 (PMC8088687; doi:10.1186/s13063-021-05283-9)
Supplement: Supplementary file 3 — Additional file 3. Ethical approval document (in Dutch). [file 13063_2021_5283_MOESM3_ESM.pdf]

MEC-U
MEDICAL RESEARCH ETHICS
COMMITTEES UNITED

Medical research Ethics
Committees United

Postbus	Postbus 2500<br>3430 EM Nieuwegein
Bezoekadres	Koekoekslaan 1 te Nieuwegein<br>088 320 8784
E-mail	info@mec-u.nl
Website	www.mec-u.nl

St. Antonius Ziekenhuis
t.a.v. drs. J.SE. Ottenhoff, ANIOS Heelkunde
Postbus 2500
3430 EM UTRECHT/NIEUWEGEIN

Betreft: besluit R19.003 Datum: 9-8-2019
NL68225.100.18

Geachte mevrouw Ottenhoff,

Hierbij ontvangt u het besluit van MEC-U over uw onderzoeksvoorstel getiteld: “*Difference in Patient Reported Outcomes at 1 Year After 6 or 8 Weeks of First Carpometacarpal Joint Distraction: a Randomized Controlled Trial*” en ons registratienummer **R19.003**.

MEC-U verleent haar goedkeuring aan het onderzoek. De goedkeuring betreft de uitvoering in de in het besluit vermelde centra.

Zie het bijgevoegde besluit voor de overwegingen bij het besluit.

MEC-U wijst u erop dat definitieve toestemming van de Raad van Bestuur van het St. Antonius Ziekenhuis nodig is voordat tot uitvoering van het onderzoek kan worden overgegaan.

Wij vertrouwen erop u hiermee voldoende te hebben geïnformeerd.

Met vriendelijke groet,
secretariaat MEC-U

BESLUIT
Primaire beoordeling.

**NL-nummer**	NL68225.100.18	**Registratienr.**	**R19.003**
**Titel onderzoek**	Difference in Patient Reported Outcomes at 1 Year After 6 or 8 Weeks of First Carpometacarpal Joint Distraction: a Randomized Controlled Trial		

Contactgegevens: drs. J.S.E. Ottenhoff (St. Antonius Ziekenhuis)
Verrichter: St. Antonius Ziekenhuis te Nieuwegein

Besluit
Medical research Ethics Committees United (MEC-U) heeft zich, op grond van artikel 2, tweede lid, sub a van de Wet medisch wetenschappelijk onderzoek met mensen (WMO), beraden over bovenstaand onderzoeksdossier.

De commissie oordeelt positief over het onderzoeksdossier uit te voeren in het volgende centrum:

- St. Antonius Ziekenhuis te Nieuwegein, drs. J.S.E. Ottenhoff (hoofdonderzoeker).

Documenten
Het oordeel is gebaseerd op de documenten die in bijlage 1 zijn vermeld.

Achtergrond
Op 14-01-2019 is het onderzoeksdossier ter beoordeling bij MEC-U ingediend. (Na ontvangst van de ontbrekende stukken op 23-01-2019, is het dossier in behandeling genomen). Het onderzoeksdossier is besproken in de vergadering(en) van 04-02-2019 en 05-08-2019 (zie bijlage 2 voor de aanwezige leden op 04-02-2019.

Op een vraagbrief en/of verzoek om aanvullende informatie vanuit MEC-U d.d. 13-02-2019 en 03-07-2019 is door de indiener gereageerd op 16-06-2019, 24-07-2019 en 07-08-2019.

Overwegingen
MEC-U is van oordeel dat aan de voorwaarden in artikel 3, eerste lid, onder a t/m m, van de WMO is voldaan. De belangrijkste vragen betroffen het protocol, het ABR formulier, de proefpersoneninformatie, vragenlijst, verzekering en onderzoeksverklaring.
De belangrijkste argumenten van de commissie om over te gaan tot een positief besluit zijn dat de vragen genoegzaam zijn beantwoord en de documenten correct zijn aangepast.

De commissie heeft de in bijlage 1 vermelde onderzoeksverklaring bekeken. Zij heeft geconstateerd dat is voldaan aan de voorwaarden in artikel 3, onderdeel f van de WMO.

De commissie is van oordeel dat het onderzoeksprotocol in een toestemmingsprocedure voorziet die overeenstemt met artikel 6, eerste en derde lid, van de WMO.

De commissie is van mening dat is voldaan aan de voorwaarden in artikel 6, vijfde t/m negende lid, van de WMO. De proefpersonen worden op gepaste, volledige en begrijpelijke wijze schriftelijk over het onderzoek geïnformeerd en over de mogelijkheid om de toestemming te allen tijde in te trekken.

Verzekeringen
MEC-U heeft geconstateerd dat is voldaan aan de verzekeringsplicht. Er is een proefpersonenverzekering afgesloten zoals bepaald in artikel 7, eerste lid, van de WMO en zoals nader uitgewerkt in het Besluit verplichte verzekering bij medisch-wetenschappelijk onderzoek met mensen 2015 (Besluit van 24 november 2014). Het onderzoek valt onder de proefpersonenverzekering van het St. Antonius Ziekenhuis.

Deelnemende instellingen: St. Antonius Ziekenhuis te Utrecht/Nieuwegein, Catharina Ziekenhuis te Eindhoven,
Diakonessenhuis te Utrecht/Zeist/Doorn, Maasstad Ziekenhuis te Rotterdam, Meander Medisch Centrum te Amersfoort/Baarn en OLVG te Amsterdam

De commissie heeft geconstateerd dat een aansprakelijkheidsverzekering is afgesloten zoals bepaald in artikel 7, negende lid, van de WMO.

Ten slotte wijst MEC-U u op de voorwaarden en verplichtingen die in bijlage 3 zijn vermeld.

Met vriendelijke groet,
b.a.

Mr. M.M. Westenbrink, ambtelijk secretaris.

Namens dr. B. van Ramshorst,
voorzitter Medical research Ethics Committees United (MEC-U).

Nieuwegein, 9-8-2019

Beroepsprocedure
Tegen dit besluit kan een belanghebbende op grond van artikel 23 van de WMO binnen zes weken na de dag waarop het besluit is bekend gemaakt, administratief beroep instellen bij de Centrale Commissie Mensgebonden Onderzoek (CCMO). Het beroepschrift dient u te adresseren aan CCMO, Postbus 16302, 2500 BH Den Haag.

Deelnemende instellingen: St. Antonius Ziekenhuis te Utrecht/Nieuwegein, Catharina Ziekenhuis te Eindhoven,
Diakonessenhuis te Utrecht/Zeist/Doorn, Maasstad Ziekenhuis te Rotterdam, Meander Medisch Centrum te Amersfoort/Baarn en OLVG te Amsterdam

Bijlage 1

Documenten

A1. Aanbiedingsbrief d.d. 13-1-2019, ontvangen d.d. 14-1-2019;
A1. Correspondentie: vraagbrief MEC-U d.d. 13-2-2019 en antwoordbrief indiener d.d. 16-6-2919;
A1. Correspondentie: vraagbrief MEC-U d.d. 3-7-2019 en antwoordbrieven indiener d.d. 24-7-2919 en 7-8-2019;
B1. ABR-formulier versie 4 d.d. 12-6-2019;
C1. Onderzoeksprotocol versie 3 d.d. 24-7-2019;
E1/2. Schriftelijke informatie voor de proefpersonen en/of voor hun wettelijke vertegenwoordigers, inclusief bijbehorende toestemmingsverklaring versie 3 d.d. 24-7-2019;
F1. Vragenlijst PROMIS PF UE;
F1. Vragenlijst PHQ en PCS;
F1. Vragenlijst MHQ;
F1. Vragenlijst patiënt demografie;
G1. WMO-proefpersonenverzekering van St. Antonius Ziekenhuis: polisnummer 624.100.023 van Centramed B.A. d.d. januari 2019;
G2. Bewijs dekking aansprakelijkheid van St. Antonius Ziekenhuis: polisnummer 626.107.129 van Centramed B.A. d.d. januari 2019;
H1. Cv van de onafhankelijke deskundige (dr. E.J. Slim);
I2. Onderzoeksverklaring van het afdelingshoofd van de afdeling Plastische Chirurgie, St. Antonius Ziekenhuis te Nieuwegein, d.d. 18-7-2019;
I3. Cv hoofdonderzoeker St. Antonius Ziekenhuis (drs. J.S.E. Ottenhoff) 2019;
I3. Certificaat herregistratie BROK projectleider St. Antonius Ziekenhuis (dr. A.B. Mink van der Molen) d.d. 27-2-2019;
K6. Brief aan huisarts (*versie toegestuurd per e-mail d.d. 16-6-2019*).

Deelnemende instellingen: St. Antonius Ziekenhuis te Utrecht/Nieuwegein, Catharina Ziekenhuis te Eindhoven,
Diakonessenhuis te Utrecht/Zeist/Doorn, Maasstad Ziekenhuis te Rotterdam, Meander Medisch Centrum te Amersfoort/Baarn en OLVG te Amsterdam

Bijlage 2

Samenstelling MEC-U
De volgende leden waren aanwezig tijdens de commissievergadering van 04-02-2019:

- dr. B. van Ramshorst, chirurg (voorzitter);
- dr. S.T.F.M. Frequin, neuroloog;
- mw. dr. S. Houterman, methodoloog;
- dr. E.F.J. van de Laar, ethicus;
- mw. A.E.H. Loth, proefpersonenvertegenwoordiger;
- dr. P.G. Noordzij, anesthesioloog;
- dr. ir. R.E.J. Sladek, klinisch fysicus;
- mw. mr. W.J. Schipper, jurist.

Bijlage 3

Voorwaarden en verplichtingen*

Geldigheid oordeel
Het positieve oordeel verliest zijn geldigheid als de inclusie van de eerste proefpersoon niet heeft plaatsgevonden binnen twee jaar nadat dit besluit is genomen.

Amendementen
Amendementen dienen ter beoordeling aan MEC-U te worden voorgelegd.

Startdatum onderzoek
MEC-U dient op de hoogte te worden gesteld van de definitieve startdatum van het onderzoek. Dat is de datum waarop de inclusie van de eerste proefpersoon plaatsvindt.

Voortgangsrapportage
Eén jaar na datum van het oordeel, en ieder jaar daaropvolgend, dient MEC-U op de hoogte te worden gebracht van de voortgang van de studie middels het formulier Voortgangsrapportage.

Geldigheid verzekering
In het geval het verzekeringscertificaat tijdens de voortgang van het onderzoek zijn geldigheid verliest, dient aan MEC-U tijdig een afschrift van een nieuw geldig certificaat te worden toegestuurd.

Melding SAE's
SAE's dienen aan MEC-U te worden gemeld.

Melding (voortijdige) beëindiging en opschorting
(Voortijdige) beëindiging en opschorting van het onderzoek dient, met redenen omkleed, te worden gemeld aan MEC-U.

Eindrapportage
MEC-U dient op de hoogte te worden gebracht van de resultaten van het onderzoek middels een eindrapport.

Termijnen en overige uitleg ten aanzien van de indiening van de verschillende documenten aan MEC-U vindt u op de website van de CCMO.
