# Supplementary material for: Can we decrease the duration of basal thumb joint distraction for early osteoarthritis from 8 to 6 weeks? Study protocol for a non-inferiority randomized controlled trial
Source: Trials. 2021 May 1;22:316. doi: 10.1186/s13063-021-05283-9 (PMC8088687; doi:10.1186/s13063-021-05283-9)
Supplement: Supplementary file 4 — Additional file 4. English translation of Add 2. [file 13063_2021_5283_MOESM4_ESM.docx]

**ENGLISH TRANSLATION OF ETHICAL APPROVAL LETTER**

**MEC**-**U**

MEDICAL RESEARCH ETHICS COMITTESS UNITED

St. Antonius Hospital

To: Drs. J.S.E. Ottenhoff, MD

Post address 2500

3430 EM Nieuwegein

Dated August 8^th^, 2019

Concerns: decision on R19.003

NL68225.100.18

Dear miss Ottenhoff,

Hereby you receive the decision of the MEC-U regarding your study protocol entitled “Patient-Reported Outcomes at 1 Year After 6 or 8 Weeks of First Carpometacarpal Joint Distraction: a Randomized Controlled Trial” with registration number **R19.003**.

The MEC-U approves the mentioned study. The approval includes conduction of the study in the mentioned medical centers. See the additional files for the considerations regarding the decision.

The MEC-U reminds you that definitive approval must be provided by the Board of Directors of the St. Antonius Hospital before conduction of the study can start.

We trust that you are properly informed with the above mentioned statements.

Kind regards,

MEC-U secretary
