# Supplementary material for: Can we decrease the duration of basal thumb joint distraction for early osteoarthritis from 8 to 6 weeks? Study protocol for a non-inferiority randomized controlled trial
Source: Trials. 2021 May 1;22:316. doi: 10.1186/s13063-021-05283-9 (PMC8088687; doi:10.1186/s13063-021-05283-9)
Supplement: Supplementary file 5 — Additional file 5. Copy of original funding document. [file 13063_2021_5283_MOESM5_ESM.pdf]

ST ANTONIUS
een santeon ziekenhuis

Aan:
Drs. J. Ottenhoff / Dr. A. Mink van der Molen

Datum:
10 januari 2020

Onderwerp:
Subsidie Fonds Geneeskundige Innovatie 2020

ST. ANTONIUS ZIEKENHUIS
T 088 - 320 30 00
www.antoniusziekenhuis.nl

Geachte mevrouw Ottenhoff en meneer Mink van der Molen,

Middels dit schrijven bevestigen wij u dat we uw project: "Gewrichtsdistractie bij jonge patiënten met duimbasisartrose" een bijdrage toekennen van € 31.000,=.

Als tegenprestatie verwachten wij van u:
- Dat u aangeeft wanneer het project daadwerkelijk aanvangt en eindigt.
- Belangrijke zaken rondom het project met ons te communiceert.
- Een artikel in de Loupe waarin u het project kort beschrijft. Op een later moment kunt u verzocht worden om resultaten te beschrijven. De redactie van de Loupe zal hierover met u contact opnemen.
- Eenmaal per jaar een tussentijds verslag van maximaal één A4.
- Een eindverslag waarin u de resultaten van uw project beschrijft.
- Dat u beschikbaarheid bent voor een mogelijke presentatie binnen het St. Antonius Ziekenhuis.

De financiële afwikkeling zal verlopen via de heer W. van der Wind, afdeling F&I (telefoon: 088 - 320 85 52). De betrokken projectleider zal een projectaanvraag moeten indienen bij de heer W. van der Wind om zo voor een juiste financiele verwerking zorg te dragen. De volgende gegevens zijn hiervoor van belang;
- Kostenplaats, waarover het project moet lopen
- Projectmanager

Na het aanmaken van een intern projectnummer, kan er op het projectnummer besteld worden.

Voor eventuele vragen kunt u terecht bij ondergetekende of één van de andere commissieleden.

Rest ons om u heel veel succes te wensen met uw project!

Met vriendelijke groet, mede namens de overige commissieleden,

Dr. Harm van Melick, uroloog
Voorzitter Commissie Geneeskundige Innovatie St. Antonius Ziekenhuis

C.c.: E. Zonnevylle
