# Supplementary material for: Can we decrease the duration of basal thumb joint distraction for early osteoarthritis from 8 to 6 weeks? Study protocol for a non-inferiority randomized controlled trial
Source: Trials. 2021 May 1;22:316. doi: 10.1186/s13063-021-05283-9 (PMC8088687; doi:10.1186/s13063-021-05283-9)
Supplement: Supplementary file 6 — Additional file 6. English translation of Add 4. [file 13063_2021_5283_MOESM6_ESM.docx]

**ENGLISH TRANSLATION OF FUNDING LETTER**

**St. Antonius Hospital**

**To:**

Drs. J. Ottenhoff / Dr. A. Mink van der Molen

**Date:**

January 10, 2020

**Concerns:**

Funding Medical Innovation Grant 2020

Dear Miss. Ottenhoff and Mr. Mink van der Molen,

Hereby we confirm that your project entitled “Thumb joint distraction for early osteoarthritis” will be granted an amount of €31.000,=.

We expect you to:

- Mention the start and end date of the project.
- Communicate important topics regarding this project with us.
- Write a paper about the project for the Loupe journal. The editorial office of Loupe will contact you about this.
- An interim report on yearly basis
- Be available for possible presentation at the St. Antonius Hospital

The financial support will be handled by Mr. W. van der Wind, Department F&I. The involved project leader must submit a project request at Mr. W. van der Wind for correct financial processing.

For any questions you can contact the chairman or one of the other members of the committee.

Good luck with your project!

Kind regards,

Dr. Harm van Melick, urologist

Chairman of the Medical Innovation Committee St. Antonius Hospital
