# Supplementary material for: Can we decrease the duration of basal thumb joint distraction for early osteoarthritis from 8 to 6 weeks? Study protocol for a non-inferiority randomized controlled trial
Source: Trials. 2021 May 1;22:316. doi: 10.1186/s13063-021-05283-9 (PMC8088687; doi:10.1186/s13063-021-05283-9)
Supplement: Supplementary file 7 — Additional file 7. Provisionally permission for re-use of figures. [file 13063_2021_5283_MOESM7_ESM.docx]

**PLEASE NOTE PERMISSION HAS BEEN OFFICIALLY REQUESTED AND WILL BE PROVIDED BY TAYLOR & FRANCIS AS STATED BELOW**

**CONCERNS**: Permission for re-use of figures

Dear Janna,

Thank you for your email,

**Figure 4 from Anne J. Spaans, L. Paul van Minnen, Assa Braakenburg & Aebele B. Mink van der Molen (2017) Joint distraction for thumb carpometacarpal osteoarthritis: a feasibility study with 1-year follow-up, Journal of Plastic Surgery and Hand Surgery, 51:4, 254-258, DOI: 10.1080/2000656X.2016.1241789**

TRIALS is published by BMC and part of Springer Nature. Taylor & Francis and SAGE are both STM members. Please note under the [STM guidelines](https://www.stm-assoc.org/2019_05_29_STM_Permissions_Guidelines_2014.pdf), [STM publishers](https://www.stm-assoc.org/membership/our-members/) are permitted to use:

- **up to three figures (including tables) from a journal article or book chapter**, but: **not more than five figures**from a whole book or journal issue/edition**;**
- **not more than six figures from an annual journal volume**; and **not more than three figures from works published by a single publisher for an article**, and **not more than three figures from works published by a single publisher for a book chapter** (and in total not more than thirty figures from a single publisher for re-publication in a book, including a multi-volume book, with different authors per chapter)
- **use single text extracts of less than 400 words from a journal article or book chapter, but not more than a total of 800 words** from a whole book or journal issue/edition

We shall be pleased to waive our fees, and to grant you **non-exclusive world rights in all languages, covering print and e-book/e-journal usage of your Work all editions**, on the condition that:

1.            The original source of publication and Taylor & Francis Ltd, are acknowledged in the caption, including a reference to the Journal’s web site: [www.tandfonline.com](http://www.tandfonline.com/)

2.            You do not license to any third party permission to reproduce this copyrighted material, in any form, and at any time.

3.            This permission does not cover any third party copyrighted work which may appear in the material requested.

**4.**            **Any alterations/adaptions to the original work must be approved by the original author(s) of the article.**

Thank you for your interest in our Journal.

With best wishes,

Lee-Ann

**Lee-Ann Anderson**– Senior Permissions & Licensing Executive, Journals

Routledge, Taylor & Francis Group

3 Park Square, Milton Park, Abingdon, Oxon, OX14 4RN, UK.

**Permissions Tel: +44 (0)20 7017 7617**

**Permissions e-mail:**[permissionrequest@tandf.co.uk](mailto:permissionrequest@tandf.co.uk)

Web: [www.tandfonline.com](http://www.tandfonline.com/)

Taylor & Francis is a trading name of Informa UK Limited, registered in England under no. 1072954

🏞🕵Before printing, think about the environment
